# Black Elder and Its Constituents: Molecular Mechanisms of Action Associated with Female Reproduction

**DOI:** 10.3390/ph15020239

**Published:** 2022-02-17

**Authors:** Adriana Kolesarova, Simona Baldovska, Ladislav Kohut, Alexander V. Sirotkin

**Affiliations:** 1 Institute of Applied Biology, Faculty of Biotechnology and Food Sciences, Slovak University of Agriculture in Nitra, 949 76 Nitra, Slovakia; ladislav.kohut@uniag.sk; 2 AgroBioTech Research Centre, Slovak University of Agriculture in Nitra, 949 76 Nitra, Slovakia; simona.baldovska@uniag.sk; 3 Department of Zoology and Anthropology, Faculty of Natural Sciences, Constantine the Philosopher University in Nitra, 949 01 Nitra, Slovakia; asirotkin@ukf.sk

**Keywords:** elderberry, rutin, anthocyanins, agglutinins, steroidogenesis, proliferation, apoptosis, reproductive biology, reproductive disorders, cancer

## Abstract

The present review summarizes the current knowledge concerning provenance, properties, physiological and therapeutic actions of elderberry and the bioactive molecules present in the plant, with emphasis on their action on female reproduction. Elderberry or black elder (*Sambucus nigra* L.) attracts attention due to its easy cultivation and high availability of bioactive compounds. Most of the available data concerning black elder’s therapeutic action are focused on its effects such as activation of immune processes and anti-inflammatory processes (cytokine production, etc.) and regulation of hormones and their receptors in cancer cells. The effects of elderberry on reproduction have been poorly investigated so far. Nevertheless, conducted studies so far demonstrate the stimulatory influence of black elder extract and its constituents, such as rutin, anthocyanins and agglutinins, on the viability and steroidogenesis of healthy ovarian cells as well as their ability to promote apoptosis and reduce the viability and proliferation of ovarian cancer cells. Furthermore, the action of black elder extract and its constituent biomolecules, such as anthocyanins and lectins, on embryogenesis and the embryonal estradiol-estradiol receptor system have also been reported. The available information, despite limitations, suggest the applicability of black elder constituents for improvement of reproductive processes in animal biotechnology, animal production and assisted reproduction, as well as for prevention and treatment of reproductive disorders (including cancer) in veterinary and human medicine.

## 1. Introduction

The search for new regulators of reproduction is important for the solution of various problems of modern society. Intensive animal production is associated with growing incidences of farm animal infertility [1]. The occurrence of reproductive disorders is currently growing in all mammalian species [2]. The inability to have children affects 10% to 20% of all couples in the modern world, which promotes the development of reproductive medicine and assisted reproduction. Subfertility affects many more people [3,4,5]. Moreover, gynecological cancers are the key cause of mortality in women [6]. Biotechnology in animal production (from artificial insemination to generation of transgenic organisms) is focused on reproductive processes; therefore, its development requires a search for new regulators and medicines affecting these processes [7].

The majority of reproductive dysfunctions have similar causes and mechanisms—the ability of various adverse environmental factors to induce oxidative stress—including the accumulation of reactive oxygen species, which are deleterious to DNA and proteins, ovarian folliculogenesis and embryogenesis. Oxidative stress can be prevented by antioxidants [4]. The most natural, accessible and inexpensive sources of antioxidants, which can affect reproductive processes and prevent numerous reproductive disorders, are plants [6,8]. Additionally, because of implications on health, there is a growing interest in the use of plant-based antioxidants in the food industry. In this regard, replacing synthetic additives with natural bioactive compounds extracted from plants has been an important strategy for food manufacturers [9,10,11].

One of the promising and widely accessible sources of antioxidants and other biologically active substances affecting reproductive and non-reproductive processes and health could be the black elderberry (*Sambucus nigra* L.). Due to its health-promoting and sensory properties, elderberry is used primarily in the food and pharmaceutical industries [12]. The popularity of this plant among food and pharma producers is growing due to its simple cultivation, high availability and high number and quantity of bioactive compounds with physiological and therapeutic properties [10,13].

## 2. Provenance and Properties

Black elderberry belongs to the *Adoxaceae* family, and its common names are elder, elderberry, black elder, European elder, European elderberry, and European black elderberry [14]. *Sambucus nigra* is a small tree or shrub, 1–8 m tall with a strong odor. The bark is brownish, with longitudinal fractures and deep grooves. The leaves are opposite, imparipinnate, with 5–7 elliptic-lanceolate, dentate leaflets. The inflorescence is an umbel with many milky-white flowers. The fruit is a shiny black-purple color, with a subspherical drupe. The plant is found in woods, clearings and hedges from sea level to mountainous elevations [15].

Black elderberry is an extremely accessible and abundant plant native to the Northern hemisphere. Their seeds are spread rapidly by birds and other animals to colonize forest edges and disturbed areas and are nowadays diffused in various habitats including subtropical regions of Asia, North Africa and North America [12,16,17]. Since the beginning of 1980s, black elderberry has been planted and commercialized in some countries in Europe, the USA, Canada, New Zealand and Chile [16]. In Europe, elderberries have been intensively used for centuries both in the food industry to produce pies, jellies, jams, ice creams, yogurts and different alcoholic beverages [17], and in folk medicines for treatment of various diseases and ailments due to their antioxidant, anticarcinogenic, immune-stimulating, antiallergic, antiviral and antibacterial properties [18]. These aspects of elderberry have recently received significant attention, especially for antioxidant capacity, as functional compounds in food applications such as natural conservatives or food supplements [19].

All parts of this plant (flower, bark, leaf and fruits) are rich sources of dietary phytochemicals, such as carbohydrates, lipids, terpenoids, flavonoids, phenolic acids, alkaloids, etc. [16,20]. The content of the essential fatty acids, such as linoleic and α-linolenic acids, is very high (approximately 39% each), and the polyunsaturated fatty acids represent 78% of the total fatty acids. The most prominent compounds present in black elderberry fruits are polyphenols, e.g., anthocyanins, with high antioxidant capacity. The major elderberry anthocyanins are cyanidin-3-glucoside and cyanidin-3-sambubioside, which are found in elderberry juice and polar extracts [21,22,23]. In addition, elderberries are a rich source of flavanols, phenolic acids and procyanidins. Elderflowers are particularly rich in flavonoids (up to 3%), such as kaempferol, astragalin, quercetin, quercetin-3-O-glucoside, rutin, isoquercitrin and hyperoside, as well as phenolic acids, gallic acid and gentisic acid [9,23,24]. The most abundant polyphenol in elderflower is the flavonoid rutin [25]. Our screening of elderflower extract confirmed that this plant represents a rich source of polyphenols, and the most prominent compound present in elderberry extracts from flowers and berries was the flavonoid rutin [26]. Additionally, other types of polyphenols like flavonol glycosides and flavonol esters are present in elderberries [27]. Other known biologically active substances in elderberry include lectins, especially agglutinins, cyanogenic glycosides, essential oils, fatty acids, organic acids, carbohydrates, vitamins and minerals [12,28,29,30,31,32,33]. Every 100 g serving of fresh berries contains vitamin B2 (65 mg), vitamin B6 (0.25 mg), vitamin C (18–26 mg), folic acid (17 mg), biotin (1.8 mg), β-carotene (0.36 mg), pantothenic acid (0.18 mg), nicotinamide (1.48 mg), potassium (288–305 mg), phosphor (49–57 mg), pectin (0.16%) and glucose and fructose (7.5%) [34].

The characteristic aroma of elderberries is a result of (E)-β-damascenone, dihydroedulan, ethyl-9-decenoate, 2-phenyl ethanol, phenylacetaldehyde and nonanal. Alcohols, esters and aldehydes are frequently identified volatile groups in elderberries. Other major secondary metabolites comprise approximately 1% triterpenes (as α- and β-amyrin, ursolic acid and oleanolic acid) and about 1% sterols (β-sitosterol, campesterol and stigmasterol). In addition, pectins, tannins and phenolic acids are found in the flowers [35,36]. Elderflowers have a strong, flowery, pleasant odor mainly due to the presence of 0.03–0.14% of essential oils. In addition, the aroma composition of elderflowers includes aldehydes, ketones, alcohols, esters, oxides, terpenes and free fatty acids [37]. The bark, leaves, seeds and raw or unripe fruits contain the cyanogenic glycoside sambunigrin, which is potentially toxic because it can release cyanide [23,38,39].

Therefore, various parts of the elderberry plant demonstrate the presence of high amounts of biologically active molecules with a wide spectrum of effects—from antioxidative and phytoestrogenic polyphenols to toxic aldehydes and glycosides. The presence of these molecules could explain the physiological and therapeutic effects of this plant, as well as the variability in its action on different targets listed below.

## 3. Physiological and Therapeutic Actions of Elderberry and Its Constituents

Elderberry is widely used in folk medicine through its pharmacological properties [40]. Currently, it presents as one of the most used medicinal plants worldwide [41]. In folk medicine, elderberry is used in the treatment of many diseases and ailments thanks to its antioxidant, anticarcinogenic, immune stimulating, antiallergic, antiviral, antibacterial [12,18,28], antidepressant and hypoglycemic properties, as well as the ability to reduce body fat and blood lipid concentrations [12]. Elderberry flowers can be used both for prevention and therapy of a wide array of diseases due to immunomodulatory [42,43], anti-inflammatory [44,45,46], antioxidant [28,47,48,49,50], antimicrobial [12,51,52] and antiviral [41,53,54] activities. In vitro experiments demonstrated the ability of elderberry extract or its constituents to suppress proliferation and viability of various cancer cell lines [25,33,55,56,57,58] and to prevent angiogenesis in tumor [55,59]. On the other hand, in some cases (estrogenic breast cancer cells and osteosarcoma cells), the biomolecule rutin found in the black elderberry did not affect cancer cell viability [60]. However, it could improve the acetic acid-induced colitis [28]. There are some reports on the neuromodulatory, particularly anticonvulsant [61] and analgesic [28,62], effects of elderberry extracts. In rats, this extract induced central depression [28], but experiments on mice showed its antidepressant activities [63].

Elderflowers have been used in traditional medicines for the management of inflammation, skin disorders such as diuretic, colds, fevers and other respiratory disturbances [28,64,65]. For example, it has the potential to ameliorate skin photoaging and inflammation [66]. Elderberry extract inhibited the infectious bronchitis virus at an early point during replication [67,68,69]. Consumption of elderberry extract has also been suggested for people with diabetic osteoporosis for improving lipid profile and reducing atherogenic risk and hyperglycemia [70]. In vitro studies suggested that elderflower extracts stimulate insulin-dependent glucose uptake [71,72,73]. It can improve functions of the cardiovascular system, improve exercise performance and mitigate the risk of cardiovascular diseases [62]. Elderberry anthocyanins can be efficient against atherosclerosis and Helicobacter pylori, a noxious pathogen responsible for various gastrointestinal disorders including duodenal ulcer and gastric cancer [55]. There is evidence for the applicability of black elderberry for the treatment of obesity [74,75]. Recently, elderberry has received significant attention from food producers due to its applicability as a natural food conservator [19]. For better visualization of the physiological and therapeutic actions of elderberry, we summarize in vitro and in vivo studies, elderberry preparation, experimental models and effects observed in Table 1.

No substantial adverse side effects of black elderberry have been reported so far [23]. Moreover, there are a growing number of reports concerning the physiological and curative actions of elderberry. Nevertheless, the majority of these effects were shown only on limited numbers of animals or in vitro experiments, whilst clinical trials are rare. Sometimes the effect of elderberry was influenced by the kind of its extract (aqueous or ethanol), indicating the influence of solvent itself or that different solvents are extracting different plant molecules [28]. The hierarchical interrelationships (primary and secondary effects) are possible; for example, the anti-obesity and antidiabetic effects of elderberry could be explained by its stimulatory action on glucose uptake and fat metabolism.

## 4. Mechanisms of Action of Elderberry and Its Constituents

### 4.1. Constituents Responsible for Particular Effects of Elderberry

It is widely accepted that elderberries’ curative capacity is due to the presence of high amounts of polyphenolic compounds, primarily flavonols, phenolic acids and anthocyanins. The presence of polyphenols, which assure the defense of plants against pathogenic microorganisms, can also explain the ability of black elderberry to suppress the activity of viruses [41,53,54] and bacteria in the body [12,51,52] and in food items [19].

Plant polyphenols are known free radical scavengers and are able to protect the human body against oxidative stress and peroxidative processes [62,76,77,78]. The strong antioxidant capacity of elderberries is probably related to the most abundant and biologically active flavonols rutin and quercetin, as well as to gallic acids [79]. Rutin (the most abundant polyphenol in elderflower) was found to inhibit the viability of human neuroblastoma [25], leukemic [56] and breast cancer [57] cells. Therefore, the anticancer activity of black elderberry can be due to the presence of rutin. On the other hand, the ability of elderberries’ anthocyanins to suppress malignant transformation of endothelioma cells has also been demonstrated [55,80]. These reports suggest the anticancer effects of several elderberry polyphenols, which could act as synergists.

There is evidence that the positive action of elderberry on vascular health is due to the presence of anthocyanins, especially cyanidin-3-O-glucoside [59,62]. Moreover, elderberry agglutinins can selectively bind to apoptotic cells, and can be involved in the execution of mitochondrial apoptosis in various cell types [81]. Finally, elderberry agglutinin can block vascular endothelial growth factor (VEGF)-induced angiogenesis [82], which plays an important role in tumor growth. These observations indicate that the pro-apoptotic action of elderberry could be due to the presence of agglutinins. The elderberry constituents responsible for other effects of this plant remain to be identified yet. 

It may, however, be noted that search for an active compound of any plant, including elderberry, is difficult because the tested commercial and non-commercial dietary supplements usually contain multiple ingredients. In addition, substantial differences are often found between labelled and actual ingredients or their amounts [23]. Furthermore, the hypothesis concerning active components of elderberry, which are responsible for a particular effect, is based usually on a similarity of the whole plant extract’s effect and its putative constituent(s). To our knowledge, the effect of the elderberry extract and the effect of its constituents have not been compared in a single experiment yet.

### 4.2. Mediators of Effects of Elderberry and Its Constituents

As mentioned above, several curative effects of elderberry could be explained by high contents of antioxidants, which can either directly eliminate free oxygen species or promote antioxidative enzymes within the cells [62,76,77,78]. Mitigation of oxidative stress and protection of genomic DNA integrity are probably the main mechanisms of elderberry’s inhibitory action on carcinogenesis, blood vessels, brain functions, inflammatory and gut microbiota [55,80], skin aging and inflammation [66].

Furthermore, the antioxidative and anticancer effects of elderberry anthocyanins are associated with the downregulation of promoters and markers of inflammation, such as monocyte chemoattractant protein-1 (MCP-1), transcription factor nuclear factor *kappa B* (NF-κB) and interleukin 8 (IL-8). Some similar signaling substances were changed during curative elderberry action on skin photoaging and inflammation. This effect was associated with a decrease in matrix metalloproteinase-1 (MMP-1) expression, secretion of inflammatory cytokines, mitogen-activated protein kinases/activator protein 1 (MAPK/AP-1) and NF-κB signaling pathways and skin inflammation. In addition, elderberry extract improved nuclear factor E2-related factor 2/heme oxygenase-1 (Nrf2/HO-1) signaling to increase oxidative defense capacity, enhanced transforming growth factor beta (TGF-β) signaling activation to promote procollagen type I synthesis and blocked extracellular matrix degradation [66]. 

The protective effect of elderberry flavonoids and pectins against the influenza virus is probably mediated by stimulating the immune system of the host through enhancing the production of inflammatory cytokines such as interleukins IL-6, IL-8 and tumor necrosis factor (TNF) [54,68,83] and the subsequent stimulation of macrophages [84]. Therefore, elderberry antioxidants could protect the target tissues via reduction of oxidative stress and the downregulation of regulators of inflammatory processes.

In addition, black elderberry anthocyanins can improve vascular health by promoting the production of nitric oxide, which in turn promotes vascular permeability and vasodilation and reduces blood pressure [62]. On the other hand, tumor anthocyanin cyanidin-3-glucoside can suppress angiogenesis via the downregulation of production of VEGF and STAT-3, a post-receptor mediator of VEGF action on vascular development [59]. Elderberry lectins can downregulate angiogenesis via a blockade of VEGF receptors [82].

Finally, elderberry can influence some physiological processes via changes in steroid hormones and their receptors. Elderflower extracts promoted estradiol release, downregulated estrogen receptor alpha and upregulated progesterone receptor expression in cultured breast cancer cells, which are involved in the control of cell proliferation [57]. The endocrine effects of elderflower could be due to phytoestrogen activity of several elderberry polyphenols, i.e., their ability to bind steroid hormone receptors and to affect the release of steroid hormones and hormone-dependent events [85].

Taken together, the available data suggest that the non-reproductive effects of black elderberry are mediated by changes in oxidative, inflammatory, and regenerative processes, as well as angiogenesis, steroid hormones and their receptors. The functional interrelationships between these processes are possible. At least, the influence of oxidative stress and antioxidants on inflammatory processes, which in turn trigger cancerogenesis/carcinogenesis, has been suggested [86]. Furthermore, the adverse effects of oxidative stress on steroidogenesis and nitric oxide have been documented [87,88]. Nevertheless, the functional interrelationships between particular signaling pathways mediating elderberry actions require further elucidation. Furthermore, various elderberry constituents could affect different targets via several mediators—this hypothesis, however, requires validation by further studies. 

## 5. Effects of Elderberry and Its Constituents on Female Reproductive Processes

### 5.1. Effect of Elderberry and Its Constituents on Ovarian Cell Viability, Apoptosis and Proliferation

Our recent in vitro study demonstrated the promising stimulatory effect of elder-berry extracts on the viability of human ovarian granulosa cells [26]. This in vitro study indicated an increase in the number of viable ovarian cells after the addition of the ex-tracts from different parts of the elderberry plant (flowers and berries). 

The role of a particular elderberry molecule responsible for its effect could be detected by comparison of the effects of the whole elderberry extract and its particular constituents. The most known biologically active constituents of elderberry are rutin, anthocyanins and agglutinins. The most abundant polyphenol in elderflower and elderberry is the flavonoid rutin [25,26]. The comparison of the effects of elderberry extract and rutin showed the similar effects for the extracts and its major component. In experiments of Sirotkin et al. [8,89], the addition of rutin was found to increase the viability of cultured porcine ovarian granulosa cells, although this effect was not associated with changes in the accumulation of markers of proliferation (accumulation of PCNA) and cytoplasmic apoptosis (Bax). In vivo experiments, like in vitro ones, demonstrated the stimulatory action of rutin on rat ovarian cell health and ovarian folliculogenesis [90,91]. 

In contrast to healthy ovarian cells, in the case of cultured cancer cells, native and hydrolyzed rutin exerted in vitro inhibitory activity against human ovarian adenocarcinomas by suppression of the viability and proliferation of OVCAR-3 ovarian carcinoma cells [92,93]. Additionally, the viability and proliferation of ovarian cancer cells were reduced by other elderberry constituents such as anthocyanins [94], glycosylated cyanidin derivatives [95] and delphinidin [96]. Another elderberry constituent, agglutinins, was reported to reduce viability and mitochondrial activity and also induce apoptosis of cultured ovarian cancer cells [33]. 

The present observations suggest the stimulatory influence of black elderberry substances on the viability of healthy ovarian cells and the ability of elderberry to promote apoptosis and reduce the viability of cultured ovarian cancer cells. These phenomena suggest that this plant can be principally useful for stimulation of healthy ovarian cells and suppression of cancer ovarian cell functions. The comparison of the action of elderberry and its constituents indicates that its action on ovarian cell viability and proliferation can be due to the presence of rutin, anthocyanins and/or agglutinins.

### 5.2. Effect of Elderberry and Its Constituents on Ovarian Cell Steroidogenesis

Our recent study demonstrated the influence of both elderberry extracts from dark purple elderberry fruits, as well as from elderflowers on steroid hormone release by cultured human granulosa cells. The release of both progesterone and 17ß-estradiol by the cells was increased after supplementation by either elderflower extract or extract from the berries of the shrub. The results of this study demonstrated the benefits of elderberry extracts prepared from different parts of plant (flowers and fruits) in the upregulation of biosynthesis of ovarian steroid hormones in vitro [26]. 

According to the above-mentioned hypothesis, the black elder constituents responsible for the elderberry’s effects on ovarian steroidogenesis are to be suggested on the basis of the similarity of effects. Such similarity in elderberry and rutin has been reported. In vivo and in vitro studies on rats demonstrated the ability of rutin to promote the plasma luteinizing hormone (LH) level, expression of ovarian follicle-stimulating hormone (FSH) receptors, steroidogenic enzymes, ovarian folliculogenesis and fecundity [90,91]. In in vitro experiments with cultured porcine granulosa cells, the addition of rutin was able to promote 17β-estradiol and testosterone, but not progesterone release [8,89,97]. It is not to be excluded that the increase in granulosa cell viability under the influence of elderberry extract could be due to the upregulation of estradiol, a known promoter of ovarian cell viability and ovarian folliculogenesis [98]. 

The current literature does not portray evidence concerning anthocyanins’ action on ovarian steroidogenesis, although the phytoestrogenic effects of anthocyanins, especially of cyanidin and delphinidin, have been reported [99,100]. The high dietary consumption of anthocyanins by humans was associated with a lower level of plasma dehydroepiandrosterone, but not of progesterone, androgens and estrogens [101]. The stimulatory action of anthocyanin cyanidin-3-O-glucoside on FSH, LH receptors and testosterone production by mice testis has been shown [102], but similar experiments on females have not been reported. 

Again, the current literature does not demonstrate the changes in ovarian steroidogenesis under influence of agglutinins. Therefore, there is no evidence so far that elderberry action on ovarian steroid hormones could be due to anthocyanins or agglutinins. On the other hand, the high similarity in stimulatory action of elderberry extract and rutin on healthy granulosa cell viability and steroidogenesis suggests that plant action on ovarian cells can be due to the presence of the most abundant plant flavonol rutin. Furthermore, the steroid-promoting effect of elderberry indicates the potential applicability of this plant and its constituent rutin for the promotion of steroid-dependent non-reproductive and reproductive events including ovarian folliculogenesis and fecundity.

### 5.3. Effect of Elderberry and Its Constituents on Embryo

Schröder et al. [57] did not find any effect of elderflower extract on the proliferation of human embryonal trophoblast cancer cells. This observation is in line with the absence of action of the main elderberry constituent rutin on the embryogenesis of *Xenopus laevis* [103] or on the viability of cultured human lung embryonic fibroblasts and human umbilical vein endothelial cells [104]. Therefore, the influence of elderflower or its constituent rutin on embryonal cells has not been detected yet. 

Other black elder constituents, such as anthocyanins [105], and here especially cyanidin (but not kuromanin) [106], were able to improve the quality and developmental capacity of porcine embryos indicating the stimulatory action of elderberry anthocyanins on embryogenesis. A similar ability to increase the viability of chicken embryonal fibroblast cells was reported for another anthocyanin, the nightshade anthocyanin [107]. 

In contrast to elderberry anthocyanin, elderberry lectins blocked receptors of vascular endothelial growth factor (VEGF) in embryonal tissue, which is responsible for embryonal angiogenesis (endothelial cell proliferation and motility) [82]. These data indicate the stimulatory action of black elderberry anthocyanin and the adverse effect of its lectins on embryogenesis. 

Schröder et al. [57] also studied the effect of elderflower extracts on the production of estradiol and estrogen receptors alpha (ER-α) using trophoblast tumor cell lines JEG-3 and BeWo. Elderflower extracts inhibited estradiol production in JEG-3 cells and prompted in BeWo cells. Furthermore, it upregulated the accumulation of estradiol receptors in JEG-3 cell lines. These observations demonstrate that black elderberry extract can affect embryonal trophoblast and its estradiol-estrogen receptor system, which could be responsible for embryo development and maintenance of gravidity. Furthermore, it indicates the black elderberry’s influence on trophoblast cancerogenesis. 

Therefore, the available publications indicate both stimulatory and inhibitory actions of elderberry extract and its constituents rutin, anthocyanins and lectins on regulators of embryogenesis, embryonal cancerogenesis and steroidogenesis. The ability of steroid hormones to promote both embryo development [108] and malignant transformation [109] is well-known. Therefore, the influence of elderberry molecules’ action on embryo and cancerogenesis through the estrogen/estrogen receptor system could be proposed. Nevertheless, understanding such interrelationships, causes in variability in elderberry molecules on various embryonal targets, their biological significance and practical applicability requires further profound studies.

## 6. Extracellular Mechanisms of Action of Elderberry and Its Constituents on Female Reproductive Processes

Although numerous signaling pathways are involved in elderberry action on non-reproductive processes, research enabled the identification of only some pathways mediating the effect of black elderberry on reproduction, and less studies have been performed to detect mediators of this plant’s action on female reproductive processes.

The direct action of the elderberry extract on reproductive organs and the ability to affect cultured ovarian [26] and embryonal trophoblast [57] cells was demonstrated. Moreover, the elderberry constituents, such as rutin [8,89,92,93], anthocyanins [94,95,96], and agglutinins [33] directly influence the viability, proliferation and apoptosis of ovarian cells. 

The ability of elderberry constituents rutin [90,91] and anthocyanins [102] on LH and FSH release indicate that they could regulate reproductive functions through the upregulation of pituitary gonadotropins. The ability of rutin to affect ovarian gonadotropin receptors [90,91] indicates that these receptors could be the next mediators of elderberry action on the ovary. 

Elderberry extract affected steroid hormones release by ovarian granulosa cells [26] and embryonal trophoblast cells [57]. The influence of elderberry molecule rutin [90,91], but not of anthocyanins and agglutinins, on ovarian steroidogenesis has also been reported. These observations suggest that steroid hormones could be one of the mediators of the black elderberry and its molecule rutin on ovarian functions, and so can promote ovarian cell viability through the stimulation of steroidogenesis [98]. Finally, the influence of elderberry extract on embryonal cancer cell estrogen/estrogen receptor systems [57], as well as the importance of this system in control of embryogenesis [108] and cancerogenesis [109], indicate that elderberry can affect embryos via estrogens and its receptors. Nevertheless, such hypotheses require direct experimental confirmation.

## 7. Intracellular Mechanisms of Action of Elderberry and Its Constituents on Female Reproductive Processes

The current literature does not contain evidence concerning the intracellular mechanisms of the whole elderberry extract action on ovarian cells; however, there are some reports concerning the key elderberry constituents (rutin, anthocyanins, agglutinins) on cultured ovarian cells and cancer cells, which could help to understand the mediators of elderberry actions on either healthy or malignant cells. 

Treatment of mice with rutin boosted ovarian follicular health and ovarian cell proliferation, increased the number of active mitochondria and the intracellular level of the antioxidant glutathione, reduced ovarian cell ROS level and markers of apoptosis (PTEN/FOXO3a) pathway and mitigated the toxic effect of cisplatin on ovarian functions [110].

The other elderberry constituents, namely anthocyanins [105] including cyanidin [106] and nightshade anthocyanin [107], activated antioxidant enzymes and reduced the contents of ROS in embryonal cells. These changes were associated with improvement of embryo quality and viability [105,106]. These reports suggest that elderberry anthocyanins can boost embryo viability via the elimination of ROS-induced oxidative stress. In cultured cancer cells, another elderberry anthocyanin, delphinidin, was able to decrease the intracellular level of ATP, changed the intracellular amount of ROS and antioxidant glutathione and decreased the mitochondrial membrane potential and mitochondrial mass indicating oxidative stress-induced apoptosis [111]. In addition, delphinidin can suppress proliferation and migration of ovarian cell carcinoma cells through blocking AKT and ERK1/2 MAPK signaling pathways [96]. On the other hand, anthocyanidins possess weak phytoestrogenic properties [99,100]. Due to these properties, they could promote proliferation of estrogen-dependent cancer cells [99]. 

A similar mechanism of action on ovarian cancer cells was reported for black elderberry agglutinin. Chowdhury et al. [33] demonstrated that the suppressive action of the black elderberry constituent on ovarian cancer cells is mediated by a cascade of events:

(1) Black elderberry agglutinin activates the signaling pathways of AKT and ERK1/2, which promotes de-phosphorylation of dynamin-related protein-1 (Drp-1).
(2) Upon its translocation to the mitochondrial fission loci, Drp-1 induces fragmentation of the mitochondrial membrane.
(3) Mitochondrial outer membrane permeabilization results in the generation of ROS and cytochrome-c release into the cytosol—the signs of mitochondrial apoptosis.
(4) These changes may result in cell cycle arrest before the G2/M phase and programmed cell death.

**Figure 1:**
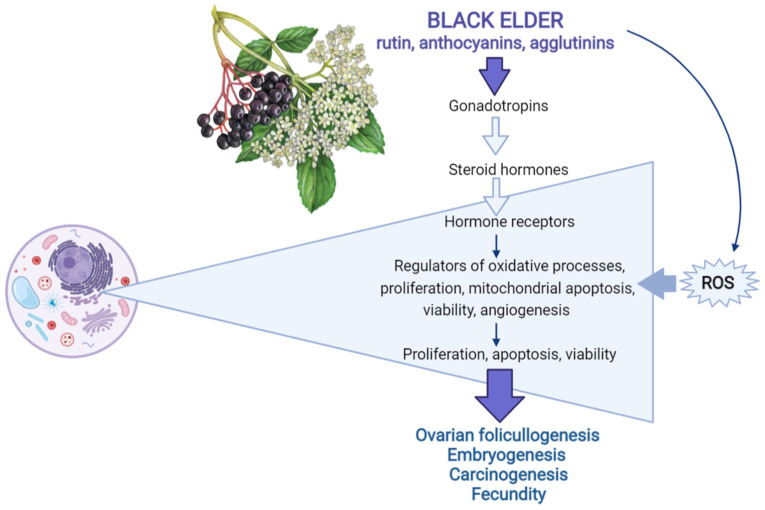
Regulators of female reproductive processes—targets of black elderberry (*Sambucus nigra* L.) and its constituents, rutin, anthocyanins and agglutinins (simplified). Explanations are provided in the text.

Results of the study also demonstrated that black elderberry and its constituent rutin could promote healthy ovarian cell functions through the upregulation of gonadotropins, gonadotropin receptors, steroid hormones, antioxidants, reduction in oxidative stress and apoptosis, which in turn improves ovarian cell viability and fecundity. In contrast, in the case of ovarian cancer cells, elderberry and its constituents, rutin, anthocyanins and agglutinin, can suppress cell viability and functions via the downregulation of the similar mechanisms—AKT- and ERK1/2-dependent intracellular signaling pathways—promoters of cell proliferation and by induction of ROS-induced cytoplasmic/mitochondrial apoptosis. Understanding the causes of opposite action of elderberry and its molecules on healthy and cancer ovarian cells via similar intracellular mechanisms require further studies. Such studies could be helpful for possible application of elderberry for stimulation of healthy ovary and reproductive processes and suppression of ovarian cancer (see below). Available evidence concerning the possible regulators of female reproductive processes affected by black elderberry and its constituents are summarized in Figure 1.

## 8. Application in Reproductive Biology and Medicine

The reported effects of black elderberry and its constituents on female reproductive processes summarized here indicate that these plant preparations could be stimulators of healthy ovarian cells and embryogenesis. It is not to be excluded that these preparations can support ovarian folliculogenesis, oogenesis, fecundity, embryogenesis and gravidity, to relieve the age-related reproductive insufficiency and menopause-related problems. As promoters of release and reception of gonadotropins and steroid hormones, as well as phytoestrogens, they could be useful in veterinary and human medicine for the treatment of disorders induced by a deficit of these hormones—age-related reproductive insufficiency, menopause-related problems, osteoporosis and polycystic ovarian syndrome. The ability of elderberry and its molecules to promote reproductive processes and fecundity could be useful in the induction of ovulation and superovulation in biotechnology in animal production and assisted reproduction. On the other hand, the ability of black elderberry substances to downregulate cell proliferation and viability of ovarian and trophoblast tumor cells indicates the potential ability of these substances for prevention, mitigation and treatment of ovarian and embryonal cancers. 

Efficiency of elderberry’s polyphenols could be increased by search of its safe and effective dosage, and the improvement of its stability, bioavailability and delivery by using nano-emulsion and nanoliposome systems, which can enhance the biological and therapeutic activity of these plant molecules [74,112]. 

Analysis of the available literature performed here shows that black elderberry contains several biologically active substances with different, sometimes opposite, actions. Some elderberry constituents could be toxic (see Section 2). Some other elderberry constituents have different chemical structures, but similar mechanisms of action (see Section 4, Section 6 and Section 7). On the other hand, the substantial differences in character, targets (see Section 5) and mechanisms of action (see Section 6 and Section 7) of various elderberry substances have been demonstrated. These facts indicate that the application of pure elderberry constituents with defined contents, amounts and effects could be more specific, controllable and efficient than the use of the whole crude plant extract. The published reports indicate that among the identified black elderberry constituents, rutin, anthocyanins (especially cyanidin-3-O-glucoside) and agglutinins could be molecules that are the most active and promising as both stimulators of reproductive functions and inhibitors of ovarian cancer. Both rutin and anthocyanins can promote gonadotropin release and reception, as well as promote ovarian cell functions. It is possible that these substances could be potentially useful as additives to gonadotropins for induction of ovarian folliculogenesis and the induction of ovulation and superovulation in animal biotechnology, assisted reproduction and medicine. On the other hand, only anthocyanins, but not rutin or agglutinins, were able to support embryogenesis. It is possible that anthocyanins could be applicable as additives to the culture medium for improvement of embryo development in in vitro embryo production. All the three mentioned elderberry constituents were shown to possess anticancer activity, and therefore all three preparations possess the potential to be useful in the management of ovarian cancer. Nevertheless, the number of reported studies is too modest to make a definite conclusion concerning elderberry constituent most suitable for particular biomedical applications.

## 9. Conclusions and Possible Direction of Future Studies

The reproductive effects of black elderberry extract and its constituents have been investigated insufficiently. Nevertheless, the published reports demonstrate the stimulatory influence of black elderberry extract and its constituents—rutin, anthocyanins and agglutinin—on steroid hormones and their receptors in ovarian and embryonal cells. The stimulatory influence of anthocyanins on embryogenesis has also been reported. On the other hand, rutin, anthocyanins and agglutinins can suppress ovarian and embryonal cancer cell functions. The effects of black elderberry and its molecules could be mediated by intracellular signaling pathways regulating cell proliferation, oxidative processes, cytoplasmic/mitochondrial apoptosis and viability, as well as by estrogen and estrogen receptors and angiogenesis. The available evidence indicates the potential usefulness of black elderberry extract and its constituents rutin and anthocyanins as biostimulators of female reproductive processes, as well as the potential applicability of whole elderberry extract, with rutin, anthocyanins and agglutinins, for the treatment of ovarian cancer and other reproductive disorders in animal biotechnology, animal production, assisted reproduction and veterinary and human medicine.

Nevertheless, the current state-of-art research invokes more queries concerning black elderberry action on female reproduction than answers. All biologically active constituents of black elderberry remain to be identified, and their biological activity remains to be investigated. The action of black elderberry extract has not been studied in in vivo experiments, whilst the reproductive effects of elderberry constituents were studied mainly in in vitro experiments and in a few animal studies. Adequate clinical studies have not been reported yet. Moreover, characteristics and mechanisms of action and biological activities of several elderberry constituents have not been compared in the same experiments yet. The conclusions concerning mechanisms of action of elderberry and its molecules are made mainly based on observations of changes in some signaling molecules after treatment, but the physiological role and functional interrelationships between particular signaling molecules remain rather hypothetical. Therefore, although the influence of black elderberry and its constituents on female reproductive processes has been demonstrated, its actions and potential applications require further studies.

## Figures and Tables

**Table 1:** Physiological and therapeutic actions of elderberry.

Therapeutic Actions	Elderberry Preparation	Experimental Model	Results	Ref.
Antimicrobial activity	Water elderberry fruit extract	*Mycoplasma mycoides subspecies capri* strain GM12, *Escherichia coli* strain DH5α and *Bacillus subtilis* strain ATCC 6051	In vitro growth inhibition of bacterial pathogens	[51]
Antiviral activity	Ethanol elderberry fruit extract Concentrated elderberry fruit juice	Madin–Darby canine kidney cells (MDCK)	Inhibition of Human Influenza A (H1N1) virus	[53,54,68]
	Concentrated elderberry fruit juice	Female BALB/c mice infected with influenza A virus	Suppression of the viral replication in the bronchoalveolar lavage fluids (BALFs); increase of the human influenza A virus (IFV)-specific neutralizing antibody in the serum; increase of secretory IgA in BALFs and feces	[54]
	Ethanol elderberry fruit extract	Vera cells	Inhibition of Infectious Bronchitis virus (IBV) by reduction in virus titers	[67]
Anti-inflammatory activity	Ethanol elderberry fruit Elderflower extract	Lipopolysaccharide (LPS)-activated cells RAW 264.7 and dendritic cells D2SC/I	Strong complement fixating activity and inhibitory effect on NO production	[44]
	Gastrointestinal digested water elderberry fruit extract	Co-cultured human intestinal epithelial cells Caco-2 and lipopolysaccharide (LPS)-activated cells RAW 264.7	Downregulation the expression of major genes of inflammatory pathway IL-1β, IL-6, TNF-α and COX-2	[46]
	Ethanol elderberry fruit extract	Human skin keratinocytes HaCaTs	Protective effect against UVB-induced skin photoaging and inflammation; suppression of UVB-induced matrix metalloproteinase-1 (MMP-1) expression and inflammatory cytokine secretion; inhibition of mitogen-activated protein kinases/activator protein 1 (MAPK/AP-1) and nuclear factor- κB (NF-κB) signaling pathways	[66]
Immuno-modulatory activity	Elderberry fruit juice	Alveolar carcinoma cells A549	Stimulation of human inflammatory cytokines IL-6, IL-8 and TNF production	[68]
	Water elderberry fruit extract	Murine-derived dendritic cells	Stimulation of *L. acidophilus*-induced IL-12 and IFN-β production	[42]
	Elderberry extract syrup Sambucol	Normal human monocytes	Stimulation of the inflammatory cytokines IL-1β, IL-6, IL-8 and TNFα production; causes a shift in the immune response to inflammation-associated Th1 responses	[43]
Antioxidant activity	Water elderberry fruit extract Ethanol elderberry fruit extract	Human intestinal epithelial cells Caco-2 and human skin keratinocytes HaCaTs	Reduction in the intracellular reactive oxygen species (ROS) production	[46,66]
	Water elderberry fruit extract	Weissberger’s biogenic oxidative system	Inhibition of oxidative degradation of hyaluronan (HA); ability to scavenge free radicals	[50]
Anticancer activity	Ethanol elderflower extract	Breast carcinoma cells MCF7	Protective effect against breast cancer by reduction of cell proliferation; inhibition of estrogen secretion, downregulation of ERα and upregulation of PR	[57]
	Butanolic elderflower extract	Bladder carcinoma cells T24 and human fibroblast cells MRC-5	Selective cytotoxic activity in cancer cells	[58]
	*Sambucus nigra* agglutinin	Epithelial ovarian adenocarcinoma cells OAW-42, p53 null OC cells SKOV3, normal epithelial ovarian cell line IOSE-364, mouse fibroblast cells NIH3T3 and lung carcinoma cells A549	Protective effect against ovarian cancer by induction of apoptosis in cancer cells and cell cycle arrest before G2/M phase; inhibition of cancer progression; mitochondrial dysfunction through increase in ROS generation and cytochrome-c release; shift of cellular respiration toward oxidative phosphorylation	[33]
Antidepressant activity	Methanol elderberry fruit extract	Male Swiss albino mice	Antidepressant potential in forced swimming test (FST) and tail suspension tests (TST)	[63]
Antidiabetic activity	Aqueous elderflower extract	Mice abdominal muscles	Increase in muscle glucose uptake, glucose oxidation and glycogenesis	[73]
	Aqueous elderflower extract	Rat pancreatic beta-cells BRIN-BD11	Stimulation of insulin secretion	[73]
	Methanol polyphenolic elderberry fruit extract	Wistar white male rats, streptozotocin (STZ)-induced hyperglycemic rats	Reduction in the body fat in diabetic rats; decrease in the lipid peroxidation level in serum	[70]
	Methanol elderflower extract	Primary porcine myotube cultures	Modulation of glucose; increase in glucose uptake	[71]
Antiosteoporosis activity	Methanol polyphenolic elderberry fruit extract	Wistar white male rats, streptozotocin (STZ)-induced hyperglycemic rats	Improvement of the bone mineral density and osteoporosis status	[70]
Anti-obesogenic activity	Anthocyanin-rich spray-dried black elderberry extract	C57BL/6 male mice, diet-induced obese mouse model	Decrease in liver weight, serum triglycerides (TAG), inflammatory markers and insulin resistance; reduction of hepatic cholesterol and lipid synthesis	[75]
	Methanol elderflower extract	Mouse embryonic fibroblast cells 3T3-L1	Activation of the peroxisome proliferator-activated receptor (PPAR) γ; stimulation of insulin-dependent glucose uptake	[72]
	Methanol elderflower extract	Primary porcine myotube cultures	Modulation of lipid metabolism; reduction of fat accumulation	[71]
Aromatase activity	Ethanol elderberry fruit Ethanol elderflower extract	Human ovarian granulosa cells HGL5	Stimulatory effect on ovarian steroidogenesis; upregulation of steroid hormone secretion	[26]
	Ethanol elderflower extract	Chorion carcinoma cell lines JEG-3 and BeWo	Inhibition of estradiol secretion and ERα upregulation	[57]

## Data Availability

No new data were created or analyzed in this study. Data sharing is not applicable to this article.
